# Novel method for the high-throughput production of phosphorylation site-specific monoclonal antibodies

**DOI:** 10.1038/srep25174

**Published:** 2016-04-29

**Authors:** Nobuyuki Kurosawa, Yuka Wakata, Tomonao Inobe, Haruki Kitamura, Megumi Yoshioka, Shun Matsuzawa, Yoshihiro Kishi, Masaharu Isobe

**Affiliations:** 1 Laboratory of Molecular and Cellular Biology, Graduate School of Science and Engineering for Research, University of Toyama, 3190 Gofuku, Toyama-shi, Toyama, 930-8555, Japan; 2 Frontier Research Core for Life Sciences, University of Toyama, 3190 Gofuku, Toyama-shi, Toyama, 930-8555, Japan; 3 Graduate School of Science and Engineering for Education, University of Toyama, Toyama-shi, Toyama, 930-8555, Japan; 4 Medical & Biological Laboratories Co., Ltd., 15-502 Akaho, Komagane, Nagano, 399-4117, Japan

## Abstract

Threonine phosphorylation accounts for 10% of all phosphorylation sites compared with 0.05% for tyrosine and 90% for serine. Although monoclonal antibody generation for phospho-serine and -tyrosine proteins is progressing, there has been limited success regarding the production of monoclonal antibodies against phospho-threonine proteins. We developed a novel strategy for generating phosphorylation site-specific monoclonal antibodies by cloning immunoglobulin genes from single plasma cells that were fixed, intracellularly stained with fluorescently labeled peptides and sorted without causing RNA degradation. Our high-throughput fluorescence activated cell sorting-based strategy, which targets abundant intracellular immunoglobulin as a tag for fluorescently labeled antigens, greatly increases the sensitivity and specificity of antigen-specific plasma cell isolation, enabling the high-efficiency production of monoclonal antibodies with desired antigen specificity. This approach yielded yet-undescribed guinea pig monoclonal antibodies against threonine 18-phosphorylated p53 and threonine 68-phosphorylated CHK2 with high affinity and specificity. Our method has the potential to allow the generation of monoclonal antibodies against a variety of phosphorylated proteins.

Protein post-translational modifications play an important role in a variety of cellular processes. Phosphorylation, the most prevalent post-translational modification of eukaryotic proteins, regulates many cellular processes, including the cell cycle, growth, and apoptosis as well as signal transduction pathways^1^. Phosphorylation sites tend to appear in clusters within relatively short segments of polypeptide chains, and increasing evidence suggests that multisite phosphorylation provides a biochemical code for achieving the intricate regulation of protein function by modulating sensitivity and threshold in controlled protein-protein interactions^2^,^3^. To understand how phosphorylation is coordinated and how individual phosphorylation patterns affect protein function, it is absolutely critical to analyze the phosphorylation status, including the sites, order and magnitude. Mass spectrometry offers an accurate and sensitive approach for mapping phosphorylation sites^4^,^5^. Nonetheless, phosphorylation site-specific antibodies remain indispensable tools for detecting, quantifying or purifying phosphorylated proteins via Western blotting, immunohistochemical staining, enzyme-linked immunosorbent assay (ELISA) and immunopurification^6^,^7^.

There are two different methods for isolating monoclonal antibodies (mAbs): animal immunization and *in vitro* display technologies. Although phage display has considerable advantages over animal immunization with regard to throughput and flexibility, the isolation of high-quality phosphorylation site-specific mAbs is hampered by a various molecular factors such as library diversity, antibody expression efficiency and the biopanning process. To overcome these difficulties, the combined use of immunization and phage display has been applied to isolate a high-affinity phospho-specific mAb from chickens^8^. Recently, Koerber *et al*. reported a powerful display technology developed by installing a natural phosphate-binding motif in a mAb scaffold, with the successful selection of 51 phospho-specific mAbs^9^. In light of the current protein engineering technology, animal immunization might appear obsolete. Nonetheless, this approach is still commonly used to produce antibodies against a variety of antigens as complementary technologies for generating different types of mAbs. Indeed, by taking advantage of the natural affinity maturation of the immune system, a variety of phosphorylation site-specific polyclonal antibodies have been generated by immunizing with phosphorylated peptides^10^,^11^. However, a polyclonal antibody comprises a mixed population of antibodies with activity that varies from animal to animal, and this activity is therefore not easily reproduced. Furthermore, attempts to produce phosphorylation site-specific mAbs by traditional hybridoma methods do not have high success, and this approach still requires exhaustive screening.

Given the expected low frequency of plasma cells (PCs) that express phosphorylation site-specific antibodies in antigen-immunized animals, the key to generating such mAbs is to isolate rare clones that actually produce the desired antibodies. We recently developed a fluorescence-activated cell sorting (FACS)-based antigen-specific plasma cell (ASPC) isolation method, which we termed endoplasmic reticulum-based identification of antigen-specific antibody-producing cells (ERIAA)^12^. By combining ERIAA and our previously proposed high-throughput single cell-based immunoglobulin gene cloning method, we achieved rapid and scalable automation for mAb generation^13^. Although ERIAA enables the generation of mAbs against highly conserved antigens via the isolation of ASPCs from a variety of animals to break immune tolerance, the potential of this technology has been limited by the inability to isolate rare ASPCs with high purity. This is because ASPC separation by ERIAA depends on weakly expressed cell surface immunoglobulins as a tag for a fluorescently labeled antigen, precluding a sufficiently strong signal on ASPCs to allow specific separation. Attempting to increase the intensity of ASPC staining with fluorescently labeled antigens, we found that intracellular labeling of paraformaldehyde (PFA)-fixed cells with a fluorescently labeled antigen together with an antibody against the immunoglobulin results in very bright signals on ASPCs, far removing them from noise. Additionally, we succeeded in amplifying full-length immunoglobulin variable (V) genes from individual fixed and intracellularly labeled cells.

The p53 protein is altered by as many as 50 individual posttranslational modifications, with N-terminal transactivation domain 1 having seven phosphorylated serine (S) residues and one threonine (T); phosphorylation at S15, T18 and S20 results in abrogation of the p53-Mdm2 interaction and increased p53 stabilization, which activates the transcriptional function of the protein^14^,^15^,^16^. CHK2 is involved in the control of cell cycle checkpoints and also participates in the transduction of DNA damage and replication stress signals. Activation of CHK2 in response to DNA damage requires phosphorylation at threonine 68 (T68), which stimulates CHK2 kinase activity^17^. Although the importance of the phosphorylation of these proteins in response to various stresses has been studied, detection of T18-phosphorylated p53 and T68-phospholyrated CHK2 have typically been performed using affinity-purified polyclonal antibodies due to the extreme difficulty in generating mAbs against epitopes containing phospho-threonine residues.

Here, we present a novel protocol that allows the development of a phosphorylation site-specific mAb by applying intracellular staining for FACS. As proof-of-principle data demonstrating the feasibility of our technology, we generated mAbs against T18-phosphorylated p53 and T68-phosphorylated CHK2.

## Results

### Full-length V gene amplification from fixed and intracellularly stained single cells

**Figure 1:**
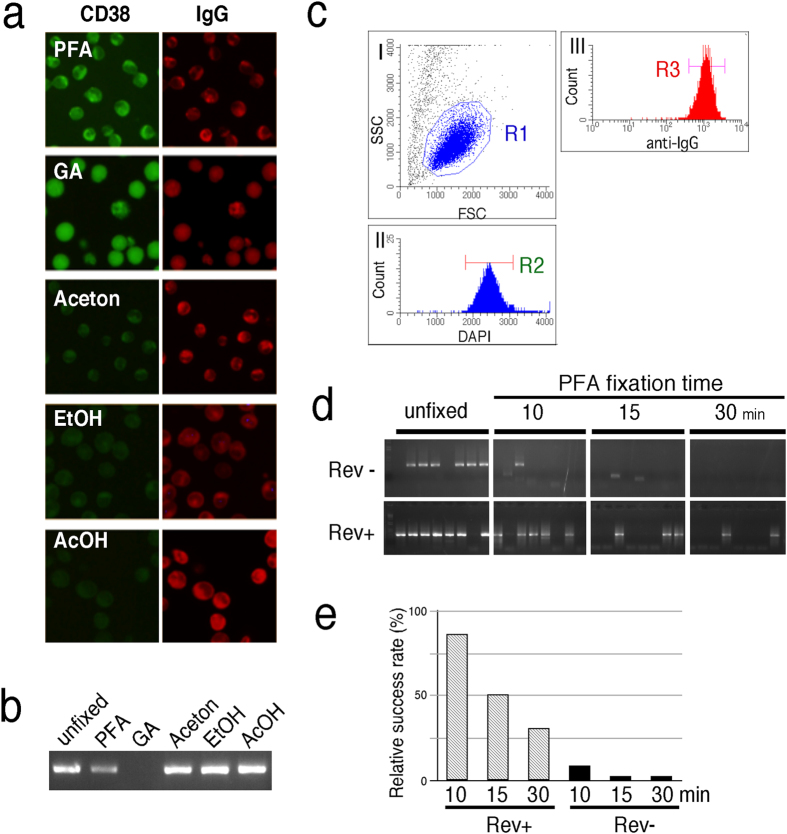
Full-length V gene amplification from PFA-fixed and intracellularly stained hybridomas at a single-cell level. (**a**) PFA-fixative preserves the antigen-binding activity of cytosolic immunoglobulin. OKT10 hybridomas spread onto APS-coated glass slides were treated with 2% paraformaldehyde in PBS (PFA), 2% glutaraldehyde in PBS (GA), 100% acetone, 100% ethanol (EtOH) or 90% ethanol-10% acetic acid (AcOH) for 20 min. After washing with PBS-0.1% Triton X-100 (PBST), the cells were stained intracellularly with DyLigh488-labeled CD38 (green) and DyLight550 anti-mouse IgG (Red). The uniform signal in GA-fixed cells is background autofluorescence caused by glutaraldehyde cross-linking. (**b**) PFA-fixative preserves the RNA integrity. OKT10 hybridomas were fixed as in (**a**) in tubes, centrifuged and suspended into Cell Lysis/Binding solution. Aliquots containing 100 cell-equivalent lysate were used for RT-PCR, amplifying 71 bp of the immunoglobulin gamma constant gene. Representative agarose gel electrophoresis is shown. (**c**) FACS gating strategy for the isolation of single hybridomas. Hybridomas were fixed with PFA, stained intracellularly with Dylight 488 anti-mouse IgG (1:250) and DAPI and then subjected to FACS analysis. Plots (I)–(III) represent the sequential gating strategy. (I) Cell debris was excluded from the R1 gate in an FSC vs SSC dot blot. (II) Single cells were selected via DAPI staining (R2). (III) A fraction of anti-IgG^high^ was defined as single hybridomas (R3). (**d**) Effects of PFA crosslinking and crosslink reversal on RNA extraction. Hybridomas fixed with PFA for various times were processed as in (**c**). R3-gated cells were single sorted into Cell Lysis/Binding solution and either left untreated (Rev−) or reverse crosslinked with proteinase K for 1 hour at 50 °C (Rev+). V genes were amplified by 5′RACE-PCR. The control consists of unfixed single hybridomas. Representative agarose gel electrophoresis of V_H_ genes is shown. (**e**) Histogram summarizing the quantitative data shown in (**d**). The results are presented as the mean percentage values of triplicates (n = 8) relative to the PCR success rate of unfixed hybridoma cells.

Terminally differentiated PCs express relatively few cell surface immunoglobulins but do contain significant quantities of immunoglobulins in the abundant rough endoplasmic reticulum combined with a well-developed Golgi apparatus. Taking advantage of these features of PCs, we and others have reported a histochemical technique for visualizing ASPCs by labeling intracellular immunoglobulins with antigens^12^,^18^,^19^,^20^. We hypothesized that a powerful method for generating mAb could be established if cells stained intracellularly with a fluorescently labeled antigen could be sorted without causing RNA degradation. To test this idea, we first attempted to determine the optimal fixative for preserving the antigen-binding activity of cytosolic immunoglobulins. The OKT10 hybridoma that produces an antibody against CD38 was used as the starting material. When the hybridomas were treated for 30 min with commonly used fixatives, permeabilized and stained intracellularly with DyLight 488-labeled CD38 and anti-mouse IgG, CD38 binding activity was only found in PFA-fixed cells; in contrast, intracellular immunoglobulin was detectable with all types of fixatives, except for glutaraldehyde (Fig. 1a). We next tested whether mRNA can be extracted from cells fixed with PFA and intracellularly stained with anti-mouse IgG (processed cells). When a 100 cell-equivalent lysate prepared from the processed cells was subjected to RT-PCR, a short amplicon (71 bp) of the mouse immunoglobulin gamma constant gene was amplified, but due to RNA-protein crosslinking, the yield was reduced compared to those treated with other fixatives (Fig. 1b). We next tested whether the full-length V gene could be amplified from the processed cell at a single-cell level. OKT10 hybridomas were fixed with PFA for 10, 15, or 30 min at 4 °C, stained intracellularly with anti-mouse IgG and 4′,6-diamidino-2-phenylindole (DAPI) and subjected to FACS for single-cell isolation (Fig. 1c). As shown in Fig. 1d, the full-length gamma heavy chain variable (V_H_) gene could not be efficiently amplified from single hybridomas via rapid amplification of 5′ cDNA ends PCR (5′ RACE PCR), regardless of the fixation time. However, 5′ RACE PCR success rates were dramatically improved when cells fixed within a short time period were treated with Protease K at 50 °C for 1 hour to reverse crosslinking (Fig. 1d,e).

**Figure 2:**
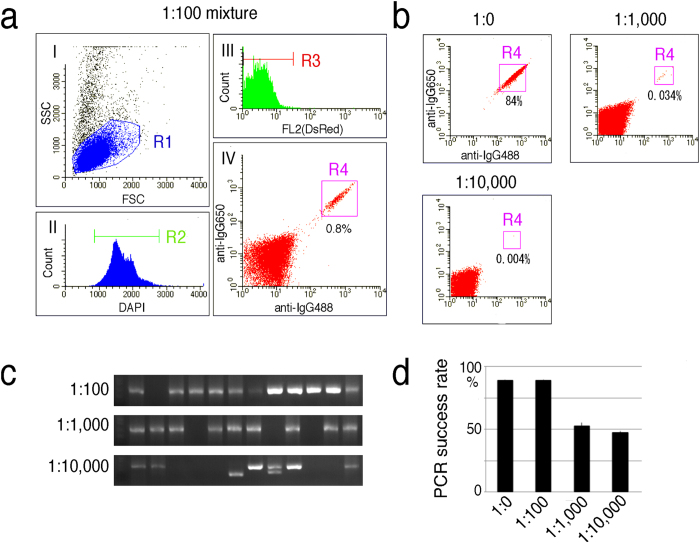
FIXAA detects low frequencies of hybridomas at less than 0.01%. (**a**) Optimization of the FACS gating strategy for the isolation of single hybridomas from an artificial mixture. Artificial samples of hybridomas spiked with Jurkat cells were fixed with PFA, stained intracellularly with DyLight 488 anti-mouse IgG, DyLight 650 anti-mouse IgG, DsRed and DAPI and then analyzed by FACS. Plots (I)–(IV) represent the sequential gating strategy. (I) Cell debris was excluded from the R1 gate in an FSC vs SSC dot blot. (II) Single cells were selected via DAPI staining (R2). (III) Nonspecifically stained cells including autofluorescent and DsRed-binding cells were excluded from the R3 gate by adjusting the FL2 threshold. (IV) A gate for hybridomas defined as DyLight 488 anti-IgG^high^, DyLight 650 anti-IgG^high^ and DsRed^negative^ was established (R4). A representative FACS graph of hybridomas mixed with Jurkat cells at 1:100 are shown. (**b**) A representative FACS graph of hybridomas spiked with Jurkat cells at 1:0, 1:1,000 and 1:10,000. The numbers indicate the percentages of cells in the gated area. (**c**) Representative agarose gel electrophoresis of V_H_ genes amplified from single cell-sorted R4-gated cells. (**d**) Histogram summarizing the quantitative data shown in (**c**). The results are presented as the mean percentage ± SD values of triplicates (n = 12).

To investigate the lower detection limit for the sensitivity of this fixed cell-based ASPC screening method (termed FIXAA), OKT10 hybridomas were serially diluted into Jurkat cells at ratios of 1:100–1:10,000, and these artificial mixtures were fixed with PFA and intracellularly stained with anti-mouse IgG, DAPI and DsRed. In an effort to eliminate background noise, DsRed was used to label damaged cells because they tend to be sticky and thus bind many types of reagents. To optimize flow cytometric gating, the FL2 threshold was adjusted to a level that minimizes the background noise caused by fixative-induced autofluorescence and non-specific DsRed-binding. As shown in Fig. 2, a fraction of hybridomas defined as anti-mouse IgG^high^ and DsRed^negative^ (R3 gate) formed a discrete population that was clearly distinguishable from Jurkat cells. Single-cell isolation of this population followed by 5′RACE PCR resulted in V_H_ gene amplification with a high success rate (87%) for a 1:100 mixture. Notably, the V_H_ gene could be reproducibly amplified from a 1:10,000 mixture with an overall success rate of 47%.

### Efficient production of monoclonal antibodies against RORγt from peptide-immunized animals by FIXAA

Retinoid-related orphan receptor gamma t (RORγt) plays a crucial role in Th17 cell differentiation^21^. Recent evidence indicates the increasing importance of RORγt in the pathogenesis of many autoimmune diseases, yet the generation of a mAb against RORγt that does not cross-react with RORγ is difficult. This is because RORγt and its splicing isoform RORγ only differ in their N-terminal extensions, consisting of 3 and 24 amino acids, respectively (Supplementary Figure 1a)^22^,^23^. Thus, we attempted to evaluate the potential usefulness of FIXAA by generating a mAb against RORγt. After immunization of guinea pigs with a KLH-conjugated RORγt peptide, lymph node cells were fixed with PFA, stained intracellularly with a DyLight streptavidin 488-conjugated RORγt peptide (RORγt-488), anti-IgG and DAPI. To eliminate plasma cells cross-reacting with RORγ, the cells were further stained with a DyLight streptavidin 550-conjugated RORγ peptide (RORγ-550) and then subjected to FACS analysis. A fraction of RORγt-specific PCs characterized as RORγt-488^high^, RORγ-550^negative^ and anti-IgG^high^ was focused into a narrow area (R4) that was sufficiently separated from rest of the cell population (Supplementary Figure 1b). A single cell-based 5′ RACE PCR of these R4-gated cells resulted in the amplification of cognate pairs of V_H_ and light chain kappa variable (V_L_) genes. DNA transfection of pairs of immunoglobulin heavy (IgH) and immunoglobulin light (IgL) genes into 293 cells resulted in the production of mAbs. Western blot screening revealed that 19 out of 20 mAbs specifically detected RORγt but not RORγ (Supplementary Figure 1c,d). By applying FIXAA, we also produced mAbs against HTLVI Tax and BCL11b from peptide-immunized rats and rabbits, indicating the usefulness of FIXAA for generating mAbs from immunized animals (Supplementary Figure 2).

### FIXAA is specialized for the generation of phosphosite-specific mAbs

**Figure 3:**
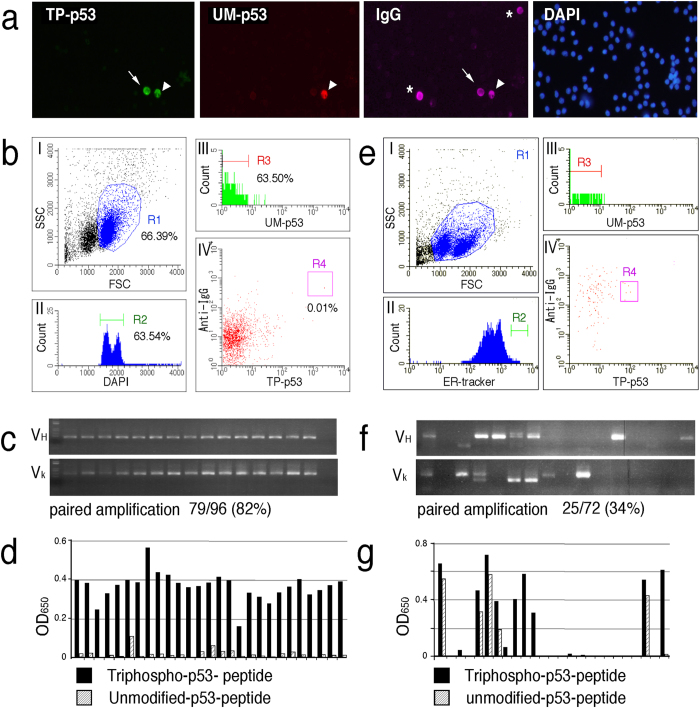
Phosphorylation site-specific mAb production by FIXAA. (**a**) Florescent microscopy images of phospho-p53-specific PCs. PFA-fixed lymph node cells from guinea pigs were intracellularly stained with TP-p53 (green), UM-p53 (red), anti-guinea pig IgG (pseudo colored pink) and DAPI (blue). Arrow, a TP-p53-specific PC. Arrowhead, a pan-p53-specific PC. Asterisks, nonspecific PCs. (**b**) FACS gating strategy for the isolation of phospho-p53-specific PCs by FIXAA. Cells treated as in (**a**) were subjected to FACS analysis. Plots (I)–(IV) represent the sequential gating strategy. (I) FSC vs SSC with gate R1 represents lymphocytes. (II) Single cells were selected via DAPI staining (R2). (III) Cells labeled with unmodified-p53 peptide were excluded from the R3 gate. (IV) The TP-p53^high^ UM-p53^negative^ and anti-guinea pig IgG^high^ fraction was defined as phospho-p53-specific PCs (R4 gate). Percentage showing the content of each cellular subpopulation among the total lymph node cells. (**c**) Representative agarose gel electrophoresis of cognate pairs of V genes amplified from single cell-sorted R4-gated cells in (**b**). The numbers indicate the percentages of paired PCR success ratios. (**d**) Triphospho-p53 peptide-binding activity of guinea pig mAbs generated by FIXAA. Cognate pairs of linear immunoglobulin heavy and light chain genes produced from single cell-sorted R4-gated cells in (**b**) were cotransfected into 293FT cells. The triphospho-p53 peptide-binding activity of each mAb analyzed by ELISA is shown. (**e**) FACS gating strategy for the isolation of phospho-p53-specific PCs by ERIAA. Live lymph node cells stained with ER-tracker, TP-p53, UM-p53 and anti-guinea pig IgG were subjected to FACS analysis. (I) FSC vs SSC with gate R1 represents lymphocytes. (II) PCs were enriched via ER-tracker staining (R2). (III) Cells labeled with unmodified-p53 peptide were excluded from the R3 gate. (IV) Phospho-p53-specific PCs were defined as ER-tracker^high^, TP-p53^medium^, UM-p53^negative^ and anti-guinea pig IgG^medium^ (R4 gate). (**f**) Representative agarose gel electrophoresis of cognate pairs of V genes amplified from single cell-sorted R4-gated cells in (**e**). The numbers indicate the percentages of paired PCR success ratios. (**g**) The triphospho-p53 peptide-binding activity of each mAb generated by ERIAA. Cognate pairs of linear immunoglobulin heavy and light chain genes produced from single cell-sorted R4-gated cells in (**e**) were cotransfected into 293FT cells. The triphospho-p53 peptide-binding activity of each mAb analyzed by ELISA is shown.

To validate the application of FIXAA for isolating ASPCs expressing phosphorylation site-specific antibodies, guinea pigs were immunized with a triphospho-p53 peptide phosphorylated at S15, T18, and S20. Fluorescence microscopy analysis of the animals’ lymph node cells stained intracellularly with anti-guinea pig IgG, triphospho-p53 peptide conjugated DyLight 488 streptavidin (TP-p53), unmodified-p53 peptide conjugated DyLight 550 streptavidin (UM-p53) and DAPI revealed phospho-p53-specific PCs intensely labeled with TP-p53 and anti-guinea pig IgG but not with UM-p53. Non-specific PCs were stained with anti-guinea pig IgG alone, and pan-p53-specific PCs or non-specifically stained cells were labeled with TP-p53, UM-p53 and anti-guinea pig IgG (Fig. 3a). Having confirmed that phospho-p53-specific PCs can be clearly distinguished from other cells via intracellular staining, we next attempted to isolate phospho-p53-specific PCs by FIXAA. Positive staining with TP-p53 and negative staining with UM-p53 revealed a clearly discrete fraction of phospho-p53-specific PCs (R4-gate) defined as anti-guinea pig IgG^high^, TP-p53^high^ and UM-p53^negative^ and comprising less than 0.01% of the original lymphocyte population (Fig. 3b). Single cell-based 5′RACE PCR of the R4-gated cells resulted in the amplification of cognate pairs of V_H_ and V_L_ genes, with a success ratio of 82% (79/96) (Fig. 3c). In addition, DNA transfection of pairs of linear IgH and IgL genes into 293FT cells resulted in the production of mAbs, 96% (47/48) of which reacted with triphospho-p53 peptide (Fig. 3d). In a parallel experiment, we also sought to isolate phospho-p53-specific PCs by ERIAA. When FACS analysis was performed with live lymph node cells stained with ER-tracker, TP-p53, UM-p53 and anti-guinea pig IgG, the phospho-p53-specific PCs (R4-gate) characterized as ER-tracker^high^, TP-p53^medium^, UM-p53^negative^ and anti-guinea pig IgG^medium^ appeared as a less discrete population (Fig. 3e). Due to the low signal-to-noise ratio, single cell-based 5′RACE PCR of R4-gated cells resulted in a low success ratio of paired V gene amplification (34%), and only 16% of the expressed mAbs reacted with the triphospho-p53 peptide (Fig. 3f,g). These results clearly demonstrate the high sensitivity and specificity of FIXAA for isolating PCs expressing phosphorylation site-specific antibodies.

**Figure 4:**
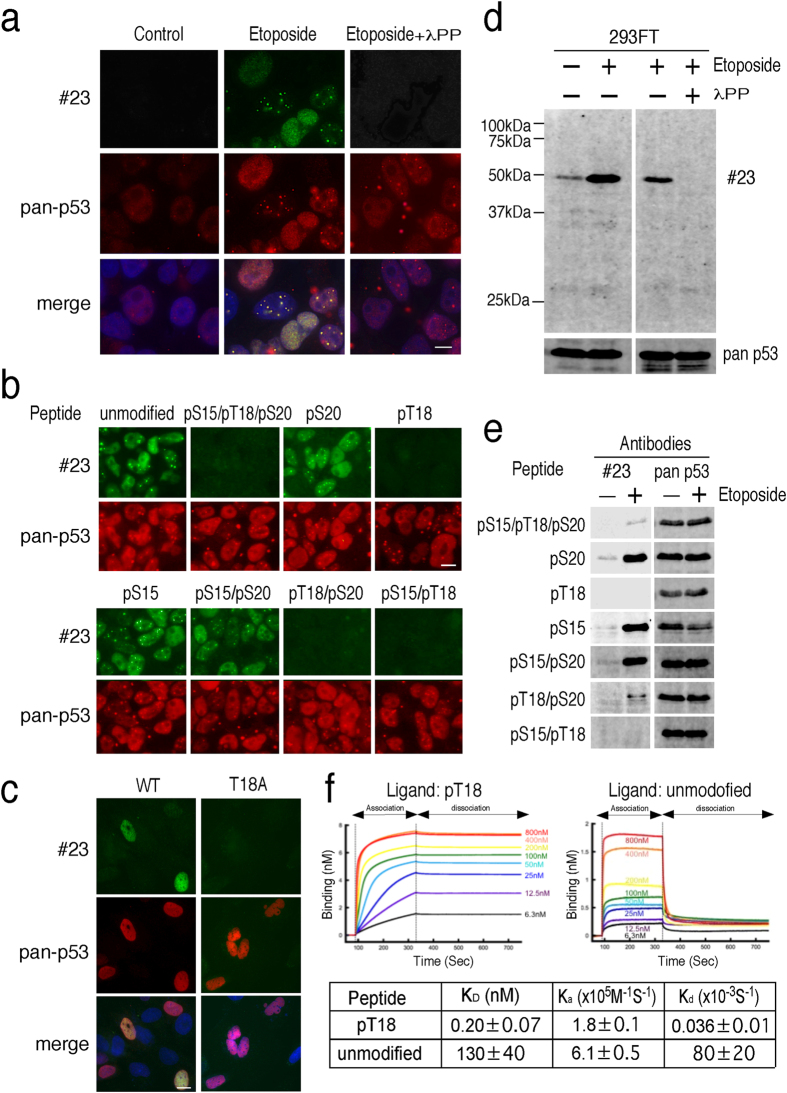
Validation of the guinea pig mAb against T18-phosphorylated p53. (**a**) Florescent microscopy images of 293FT cells stained with #23. Etoposide-treated or untreated 293FT cells were fixed, incubated with and without λPP and stained with #23 (green) and anti-pan p53 (red) antibodies. (**b**) Peptide competition experiments performed by immunofluorescence assay. The indicated blocking peptides (10 μM) were preincubated with #23 (green) and anti-pan p53 (red) antibodies. The antibody mixtures were applied to etoposide-treated 293FT cells. (**c**) Fluorescent microscopy images of Saos-2 cells expressing either WT or phosphorylation-dead mutants (T18A). Saos-2 cells electroporated with p53 plasmids were cultured for 24 hours with 100 μM etoposide, fixed and stained with #23 (green) and anti-pan p53 (red) antibodies. Scale bar, 10 μm. (**d**) Immunoblot validation of #23. An extract of etoposide-treated 293FT cells was incubated with and without λPP and probed with #23 and pan-p53 antibodies. (**e**) Peptide competition experiments performed by Western blot assay. The indicated blocking peptides (10 μM) were preincubated with #23 and anti-pan p53 antibodies. (**f**) Dose response curves for binding of #23 to the p53 peptide phosphorylated at T18. Biotinylated p53 peptides were immobilized on streptavidin sensors, and #23 was allowed to bind peptides at different dilutions. Antibody binding was measured using a FortéBio BLItz instrument. Three readings at different antibody concentrations were used for each peptide.

We next attempted to identify a mAb that binds to the p53 peptide phosphorylated at T18 (pT18) because mAbs against T18-phosphorylated p53 have yet to be described. Among 51 mAbs screened by ELISA, we found five clones that specifically bound to pT18. To test the specificity of these isolated clones, we performed immunofluorescence antibody screening and found that clone 23 (#23) reacted strongly with the nuclei of etoposide-treated 293FT cells. A signal for #23 was barely visible in control 293FT cells, whereas a signal for the pan-p53 antibody was predominantly detectable in nuclei. Lambda protein phosphatase (λPP) was applied to the etoposide-treated 293FT cells, which resulted in the loss of #23 labeling, though the pan-p53 antibody signal was not affected (Fig. 4a). The specificity of this mAb for p53 phosphorylated at T18 was confirmed by peptide competition assays, in which #23 was preincubated with either non-phosphorylated or a series of phosphorylated p53 peptides. Although incubation with either non-phosphorylated or peptides phosphorylated at sites other than T18 did not affect the detection of p53 localization in etoposide-treated 293FT cells, incubation with peptides with phosphorylation at T18 did cause an almost complete loss of reactivity (Fig. 4b). To further validate the specificity of the mAb, we carried out immunofluorescent analysis of the p53-null cell line Saos-2 transiently expressing either wild-type p53 (WT) or a T18 phosphorylation-dead alanine point mutant (T18A). As shown in Fig. 4c, #23 reacted strongly with some but not all of the nuclei of Saos-2 cells expressing WT. No signal was observed in cells expressing the T18A mutant. These data show that #23 specifically recognizes p53 phosphorylated at 18T. We also performed a Western blot analysis with total 293FT cell lysate; #23 identified a single band in etoposide-treated cells that comigrated with the band produced by the anti-pan-p53 antibody, and treatment of the cell lysate with λPP demonstrated the specificity of the #23 antibody for the phosphorylated protein (Fig. 4d). Furthermore, preincubation of the mAb with pT18, but not peptides without phosphorylation at T18, abolished immunoreactivity (Fig. 4e). Finally, #23 exhibited a dissociation constant (K_D_) value of 0.2 nM toward the pT18 peptide (Fig. 4f).

CHK2 is involved in the control of cell cycle checkpoints and also participates in transducing signals of DNA damage and replication stress. CHK2 activation in response to DNA damage requires phosphorylation at threonine 68 (T68), resulting in DNA damage-dependent nuclear foci^24^. To demonstrate the usefulness and reproducibility of our method for efficiently producing phosphorylation site-specific mAbs, we generated mAbs against T68-phosphorylated CHK2 and examined whether the isolated mAbs can detect T68-phosphorylated CHK2. Among 17 mAbs screened, #34 specifically detected CHK2 in a phosphorylation-dependent manor. The mAb exhibited a high affinity toward the pT68 peptide with K_D_ value of 0.08 nM (Supplementary Figure 3). These data clearly demonstrate that our method can efficiently produce high-quality mAbs against phosphorylated proteins.

## Discussion

Antibodies offer a platform for the analysis of various post-translational modifications^25^,^26^, and the need for modification-specific antibodies continues to grow with increasing types of modification per protein. However, highly specific antibodies against post-translationally modified proteins are difficult to produce by conventional methods^9^. To isolate rare ASPCs that express phosphorylation site-specific antibodies from animals, we developed a new FACS-based ASPC separation method, FIXAA, which targets abundant intracellular immunoglobulin as a tag for fluorescently labeled antigens. Analysis of artificial samples of hybridomas spiked with Jurkat cells demonstrated a lower detection limit for FIXAA sensitivity of less than 0.01%. FIXAA is not only able to isolate rare PCs with high purity, but by employing a subtraction step that eliminates PCs expressing antibodies against undesired antigens, it is also able to specifically isolate PCs expressing RORγt-specific antibodies that do not cross-react with RORγ. FIXAA is particularly suited for generating mAbs against a phosphorylated protein by removing PCs that react with an unmodified peptide. We achieved phospho-peptide-specific PC isolation from guinea pig lymph node cells with high purity and succeeded in generating mAbs against T18-phosphorylated p53 and T68-phosphorylated CHK2.

Although FIXAA enables the isolation of ASPCs with high specificity and sensitivity, the technique requires membrane permeabilization to expose the intracellular immunoglobulins to the fluorescently labeled antigens, which also allows RNases access to RNA. Thus, FIXAA requires stringent precautions against endogenous and exogenous RNases, including the cleaning of the FACS sample line, the inactivation of cellular RNases with PFA, the use of RNase inhibitors in the incubation steps and the preparation of RNase-free antigen. With respect to antigens, chemically synthesized peptides are well suited for FIXAA because they can be prepared under RNase-free conditions. Another limitation of FIXAA is the difficulty in amplifying full-length V genes from PFA-fixed cells because the PFA-fixation process results in cross-linkage between RNA and proteins, making RNA more recalcitrant to extraction^27^. PFA also causes the covalent modification of RNA by mono-methylol, which impairs the reverse transcriptase reaction^28^. Although several attempts have been made to address these problems, successful PCR amplification has been limited to short targets^29^,^30^,^31^,^32^. We demonstrated that full-length V genes could be reproducibly amplified by 5′RACE PCR from fixed and intracellularly stained single cells. Important aspects for the success of our procedure are as follows: (1) optimized fixation and protocols that reverse crosslinking to provide minimally modified RNA; (2) a single cell-based automatic cDNA synthesis system that enables microscale mRNA purification, reverse transcription and 3′ homopolymer-tailing; (3) abundantly expressing immunoglobulin genes in PCs might also facilitate full-length V gene amplification from fixed cells. We envisage using two ASPC isolation methods, ERIAA and FIXAA, for the generation of mAbs: ERIAA for the isolation of relatively abundant ASPCs with roughly purified antigens; FIXAA for the isolation of rare ASPCs with RNase-free antigens.

Taken together, this novel method provides a significant advantage over conventional animal immunization techniques and is particularly well suited for generating mAbs against a variety of phosphorylated proteins using chemically synthesized peptides.

## Materials and Methods

### Materials

Paraformaldehyde, Phosphatase Inhibitor Cocktail 3, NaF, sodium pyrophosphate, Na_3_VO_4_ and other chemicals and reagents were purchased from Sigma-Aldrich (http://www.sigmaaldrich.com). Cell Lysis/Binding buffer, Dynabeads Oligo(dT)_25_, recombinant CD38 and DyLight Fluor Labeling Reagents were purchased from Thermo Fisher Scientific (http://www.thermoscientific.com). Restriction enzymes and lambda protein phosphatase were purchased from New England BioLabs (http://www.nebj). Blocking One-P and Chemi-Lumi One Super were purchased from Nacalai Tesque (http://www.nacalai.co.jp). Synthetic peptides and the anti-pan-CHK2 antibody (DCS-273) were purchased from Medical & Biological Laboratories (https://ruo.mbl.co.jp). The sequences of the peptides used in this work are listed in Table 1. OKT10 hybridoma and Jurkat cells were purchased from ATCC. CD38 was labeled with DyLight Fluor Labeling Reagents and purified using Bio-Gel columns (BIO-RAD, http://www.bio-rad.com). DyLight-labeled goat secondary antibodies against IgG were purchased from Abcam (http://www.abcam.com). IRDye800CW Donkey Anti-Guinea pig IgG (H + L) and IRDye680LT Donkey Anti-Rabbit IgG(H + L) were purchased from LI-COR Biosciences (http://www.licor.com/). Rabbit polyclonal anti-pan-p53 antibody (SC-6243) was purchased from Santa Cruz Biotechnology Inc (http://www.scbt.com). DsRed protein was produced in *Escherichia coli* BL21 and purified using Superflow Ni-NTA columns (Qiagen, http://www.qiagen.com), as described previously^12^. Female Hartley guinea pigs were purchased from Japan SLC, Inc. (http://jslc.co.jp). Hela cells, Saos-2 cells and cDNAs encoding p53 were provided by the RIKEN BRC (http://ja.brc.riken.jp/) through the National Bio-Resource Project of the MEXT, Japan^33^.

### Cell culture

293FT and Hela cells were cultured in IMDM medium containing 10% FBS and were either untreated or treated with 20 μM etoposide for 12 hours or with 5 μM camptothecin for 1 hour. OKT10 hybridomas and Saos-2 cells were grown in RPMI medium containing 10% FBS.

### Lymph node cell preparation

All animal studies were approved by the Committee for Laboratory Animal Care and Use at University of Toyama and the experiments were carried out in accordance with the approved guidelines.

RORγt peptide (MRTQIEVIPC), triphospho-p53 peptide (CPLpSQEpTFpSDLWKLLPENNK-biotin) and CHK2 peptide phosphorylated at T68 (CGTLSSLETVSpTQELYSIPEDK-biotin) were conjugated to keyhole limpet hemocyanin (KLH) via the C-terminal cysteine residue using Imject Maleimide-Activated mcKLH, respectively (Thermo Fisher Scientific). Guinea pigs were immunized four times intramuscularly at the tail base with 200 μl of a 50:50 water-in-oil TiterMax Gold adjuvant emulsion containing the peptide conjugated to KLH. At 1 week after the final immunization, the iliac lymph nodes were surgically removed and used for the isolation of ASPCs^12^.

### Isolation of ASPC by FIXAA

To inactivate RNases, the FACS sample lines were rinsed with 0.1% sodium hypochlorite followed by RNase-free water. Prior to use, DyLight-conjugated secondary antibodies were diluted (1:250) in PBS-0.1% TritonX100 (PBST) containing 0.01% diethylpyrocarbonate (DEPC). Cells (1 to 10 × 10^6^) were washed with PBS, suspended in 1 ml ice-cold 2% PFA-PBS and incubated for 8 minutes on ice. The cells were precipitated by centrifugation (2 minutes at 700 × g at 4 °C) and resuspended with 250 μl of intracellular staining solution composed of fluorescently labeled antigen (0.1 μg), DyLight-conjugated secondary antibodies, DsRed (0.1 μg) and RNaseOUT (400 units). For the isolation of phosphorylation site-specific PCs, biotinylated peptide (1.6 μM) and biotinylated unmodified peptide (1.6 μM) were conjugated with DyLight streptavidin 488 (0.4 μM) and DyLight streptavidin 550 (0.4 μM), respectively, for 30 minutes at room temperature. An aliquot of 25 μl of each peptide solution was combined in 200 μl of PBST containing 10 μM biotin, goat anti-guinea pig IgG (DyLight 650) and RNaseOUT (400 units) and used as an intracellular staining solution. After incubation for 15 minutes on ice with the intracellular staining solution, the cells were then diluted with ice-cold 3 ml PBS containing 1 μM of DAPI and analyzed by FACS. Single-cell sorting and data analysis were performed using a JSAN flow cytometer equipped with an automatic cell deposition unit (http://baybio.co.jp). The channels used were as follows: anti-IgG in the FL-1 or FL-5 channel, phosphorylated peptide in the FL-1 channel, unmodified peptide or DsRed in the FL-2 channel, and DAPI or ER-tracker in the FL-7 channel.

### Molecular cloning of V_H_ and V_L_ genes from single cells and recombinant antibody expression

V_H_ and V_L_ genes were amplified from single cells as previously described, with some modifications^11^. Single cells were sorted into each well of U-bottom 96-well plates containing 20 μl Cell Lysis/Binding solution with 30 μg oligo-(dT)_25_ magnetic beads and Protease K (100 μg/ml). Reverse crosslinking was performed at 50 °C for 1 hour. The magnetic beads were concentrated in 3 μl Cell Lysis/Binding solution and subjected to 3′-end homopolymer-tailed cDNA synthesis, as described previously^12^. The V_H_ and V_L_ genes were amplified by 5′ RACE PCR using the 3′-end homopolymer-tailed cDNA fragments as the templates. The primers used were essentially the same as described before. The PCR-amplified V gene fragments were joined to their respective DNA cassettes to build linear immunoglobulin heavy and light chain genes by TS-jPCR, as described previously^13^. The PCR-amplified V gene fragments were subcloned into a mammalian expression vector by TS-homologous recombination^34^. The cognate pairs of immunoglobulin heavy and light chain genes were cotransfected into Expi293 cells according to the manufacturer’s protocol. The cell culture supernatant was assayed by ELISA for antigen-binding activity at 3 days after transfection. Briefly, biotinylated peptides (0.1 μg) were immobilized on avidin-coated 96-well plates (Sumitomo Bakelite, https://www.sumibe.co.jp). The culture supernatant (25 μl) was mixed with 25 μl of PBS containing phosphatase inhibitors (Phosphatase Inhibitor Cocktail 3, 2 mM NaF, 2 mM sodium pyrophosphate, 2 mM Na_3_VO_4_ and 1 mM EDTA); the mixture was then transferred to the peptide-coated plate and incubated at 4 °C for 3 hours. After washing the plate with PBS, the bound mAbs were detected with HRP-conjugated secondary antibodies. Recombinant mAbs were purified with Byzen Pro protein A (Nomadic Bioscience Co., Ltd, http://www.nomadicbio.com). The primers used for amplifying the mouse immunoglobulin gamma constant gene were 5′-TGCGCATGCCTGGTCAAGGGCTA-3′ and 5′-CTGGACAGGGATCCAGAGTTCCA-3′.

### Plasmid construction

p53 variants with residues replaced by alanine at positions S15, T18 and S20 were generated by QuikChange Site-Directed Mutagenesis Kit (Agilent Technologies). The PCR products were subcloned into the pEF/myc-His vector (Thermo Scientific). Sequencing (ABI 3730 XL Sequencer) was carried out to confirm all mutations. The plasmids were introduced into Saos-2 cells using CUY21 SC Square Wave Electroporator (Nepa Gene http://www.nepagene.jp/index2.html).

### Immunofluorescence analysis

Cells grown on propyltriethoxysilane-coated glass slides (Matsunami Glass, http://www.m-osaka.com) were fixed for 30 minutes with 4% paraformaldehyde-PBS, washed 3 times with PBS, and blocked with Blocking One-P for 30 minutes. Phosphorylation site-specific and pan-p53 antibodies were diluted with blocking buffer containing phosphatase inhibitors (Phosphatase Inhibitor Cocktail 3, 2 mM NaF, 2 mM sodium pyrophosphate, 2 mM Na_3_VO_4_ and 1 mM EDTA) and incubated with the cells overnight at 4 °C. The cells were washed with PBST, incubated with a 1:500 dilution of DyLight 488 goat anti-guinea pig secondary antibody and DyLight 594 goat anti-rabbit secondary antibody in blocking buffer for 1 hour at room temperature, washed with PBST, and mounted with ProLong Gold Antifade Mountant (Thermo Fisher Scientific). For phosphatase treatment, cells were fixed with 4% PFA, permeabilized with PBST and then incubated with λPP (500 unit/50 μl) for 8 hours at 30 °C. The cells were then washed and immunostained as described above. Microscopy images with the same exposure settings were taken of immunostained cells with and without phosphatase treatment. The images were captured using an Olympus IX71 fluorescence microscope equipped with a SPOT RT3 digital microscopy camera (SPOT Imaging Solutions, http://www.spotimaging.com/) and analyzed using 2D Deconvolution MetaMorph software (Molecular Devices, http://www.moleculardevices.com) or a Leica TCS SP8 Confocal Laser Scanning Microscope (http://www.leica-microsystems.com).

### Western blotting

Cell lysates were prepared by solubilizing cells in RIPA buffer (25 mM Tris-HCl pH 7.6, 150 mM NaCl, 1 mM Na_2_EDTA, 1 mM EGTA, 1% NP-40, 1% sodium deoxycholate) containing phosphatase inhibitors and cleared by centrifugation at 13,000 rpm for 30 minutes at 4 °C. For phosphatase treatment, cells were lysed in 100 mM NaCl, 50 mM Tris HCl, 2 mM DTT, 1 mM MnCl_2_ pH7.5, 0.1% NP40 for 30 minutes on ice. Protein extracts (200 μg) were incubated with solvent or 2,000 units of *λ*PP at 30 °C for 4 hours prior to analysis by Western blotting. Western blots were imaged using Odyssey Infrared Imaging System (LI-COR Biosciences).

### Peptide competition

The #23 and pan-p53 antibodies were first diluted in blocking buffers containing phosphatase inhibitors and then mixed with 10 μM peptides for 30 minutes at room temperature. All antibody solutions were subsequently used for immunofluorescent analysis and Western blotting as described above.

### Antibody affinity and kinetic assay

Antibody binding was measured using a FortéBio BLItz instrument, and the dissociation constant was analyzed using Forte Bio’s analysis software (http://www.fortebio.com). Biotinylated peptides (1.5 nM) were immobilized on streptavidin biosensors. After a baseline was established in kinetic buffer, the sensors were applied to various antibody concentrations in kinetic buffer for 240 s. Dissociation was measured in kinetic buffer for 450 s. For each subsequent run, the biosensors were regenerated by repeated washing with 10 mM glycine (pH 2.0). All measurements were performed at room temperature. Binding data at different antibody concentrations were globally fit to a simple 1:1 interaction model.

## Additional Information

**How to cite this article**: Kurosawa, N. *et al*. Novel method for the high-throughput production of phosphorylation site-specific monoclonal antibodies. *Sci. Rep.* **6**, 25174; doi: 10.1038/srep25174 (2016).

## Supplementary Material

Supplementary Information

## Figures and Tables

**Table 1:** Peptides used in this study.

Name	Sequence	Application
pS15	PLpSQETFSDLWKLL	Competition
pT18	PLSQEpTFSDLWKLL	Competition
pS20	PLSQETFpSDLWKLL	Competition
pS15/pT18	PLpSQEpTFSDLWKLL	Competition
pT15/pS20	PLpSQETFpSDLWKLL	Competition
pT18/pS20	PLSQEpTFpSDLWKLL	Competition
pS15/pT18/pS20	PLpSQEpTFpSDLWKLL	Competition
Unmodified p53	PLSQETFSDLWKLL	Competition
Biotinylated unmodified p53	PLSQETFSDLWKLLPENNK(biotin)	FIXAA, ELISA, Affinity
Biotinylated triphospho-p53	CPLpSQEpTFpSDLWKLLPENNK(biotin)	Immunization, FIXAA, ELISA
Biotinylated pT18 p53	PLSQEpTFSDLWKLLPENNK(biotin)	ELISA, Affinity
Biotinylated pT68 CHK2	CGTLSSLETVSpTQELYSIPEDK(biotin)	Immunization, FIXAA, ELISA, Competition, Affinity
Biotinylated unmodified CHK2	CGTLSSLETVSTQELYSIPEDK(biotin)	FIXAA, ELISA, Competition, Affinity
