# Supplementary material for: Novel method for the high-throughput production of phosphorylation site-specific monoclonal antibodies
Source: Sci Rep. 2016 Apr 29;6:25174. doi: 10.1038/srep25174 (PMC4850396; doi:10.1038/srep25174)
Supplement: Supplementary Information [file srep25174-s1.pdf]

Novel method for the high-throughput production of phosphorylation site-specific monoclonal antibodies.

Nobuyuki Kurosawa, Yuka Wakata, Tomano Inobe, Haruki Kitamura, Megumi Yoshioka, Shun Matsuzawa, Yoshihiro Kishi and Masaharu Isobe

Supplementary figure legends

Supplementary figure 1. Efficient generation of RORγt mAbs by FIXAA.
(a) Alignment of the amino acid stretch encompassing the N-terminal region of the RORγ isoform. Underlining indicates the position of the peptide used for immunization. **(b)** FACS gating strategy for the isolation of RORγt-specific PCs by FIXAA. Iliac lymph node cells prepared from guinea pigs immunized with the KLH-conjugated RORγt peptide (MRTQIEVIPC) were fixed with PFA and intracellularly stained with the DyLight streptavidin 488-conjugated RORγt peptide (RORγt-488), DyLight streptavidin 550-conjugated RORγ peptide (HTQIEVIPC) (RORγ-550), anti-IgG and DAPI. Plots (I)–(IV) represent the sequential gating strategy. (I) FSC vs SSC with gate R1 represents lymphocytes. (II) Single cells were selected via DAPI staining (R2). (III) Cells labeled with RORγ-550 were excluded from the R3 gate. (IV) The RORγt-488^{high} RORγ-550^{negative} and anti-guinea pig IgG^{high} fraction was defined as RORγt-specific PCs (R4 gate). **(c)** Representative agarose gel electrophoresis of cognate pairs of V genes amplified from single cell-sorted R4-gated cells in (b). **(d)** Immunoblot validation of anti RORγt mAbs. An extract of Myc-tagged RORγ- or RORγt-transfected 293FT cells was subjected SDS-PAGE and subjected to Western blot analysis with the anti-RORγt mAbs generated by FIXAA. Most of the mAbs reacted specifically with RORγt.

Supplementary figure 2. FIXAA enables production of mAbs from peptide-immunized animals. **(a)** Confocal microscopy imagines of an anti-Tax mAb generated by FIXAA. Antibodies were produced by immunizing rats with a KLH-conjugated Tax peptide (CEKEADDNDHEPQI). MT4 cells expressing Tax were stained with a rat anti-Tax mAb (#5-2), revealing Tax localization to pericentrosome and nucleus. The centrosome was stained with an anti-γ-tubulin antibody (red). **(b)** Western blot analysis of extracts from MT4 cells probed with #5-2. This antibody detects a single band of endogenous Tax. **(c)** Western blot analysis of extracts from Jurkat cells using an anti-BCL11B mAb (#3-2) generated by FIXAA. Antibodies were produced by immunizing a rabbit with a human BCL11B peptide

(SRRKQGNPQHLSQRELITPEADHVEAAILEEDEGLEIEE). #3-2 detects a single band of endogenous BCL11B. **(d)** Confocal microscopy imagines of an anti-BCL11B mAb (#6-2). Jurkat cells expressing BCL11B were stained with #6-2, revealing BCl11B localization to nuclear speckles (green). The cytosol is stained with an anti-α-tubulin antibody (red).

Supplementary figure 3. Generation of a mAb against T68-phosphorylated CHK2.
(a) FACS gating strategy for the isolation of T68-phosphorylated CHK2-specific PCs by FIXAA. Iliac lymph node cells prepared from guinea pigs immunized with the KLH-conjugated CHK2 peptide phosphorylated at T68 (pT68-CHK2) were fixed with PFA and intracellularly stained with the DyLight streptavidin 488-conjugated pT68peptide (pT68-CHK2-488), DyLight streptavidin 550-conjugated unmodified CHK2 peptide (UM-CHK2-550), anti-IgG and DAPI. Plots (I)–(IV) represent the sequential gating strategy. The pT68-CHK2-488^{high} UM-CHK2-550^{negative} and anti-guinea pig IgG^{high} fraction was defined as T68-phosphorylated CHK2-specific PCs (R4 gate). **(b)** pT68-CHK2-binding activity of guinea pig mAbs generated by FIXAA. Cognate pairs of linear immunoglobulin heavy and light chain genes produced from single cell-sorted R4-gated cells in (a) were cotransfected into 293FT cells. The pT68-CHK2-binding activity of each mAb analyzed by ELISA is shown. **(c)** Confocal microscopy imagines of camptothecin-treated or untreated 293FT cells stained with a mAb (#34; green) and the anti-pan-CHK2 antibody (red). **(d)** Confocal microscopy imagines of camptothecin-treated Hela cells were incubated with indicated blocking peptides (10 μM) or λPP and stained with #34 (green) and the anti-pan-CHK2 antibody (red). **(e)** Dose response curves for binding of the mAb #34 to the pT68-CHK2. #34 exhibited a dissociation constant (K_D) of 0.08 nM toward the pT68-CHK2 peptide.

Supplementary figure 1. Efficient generation of RORγt mAbs by FIXAA

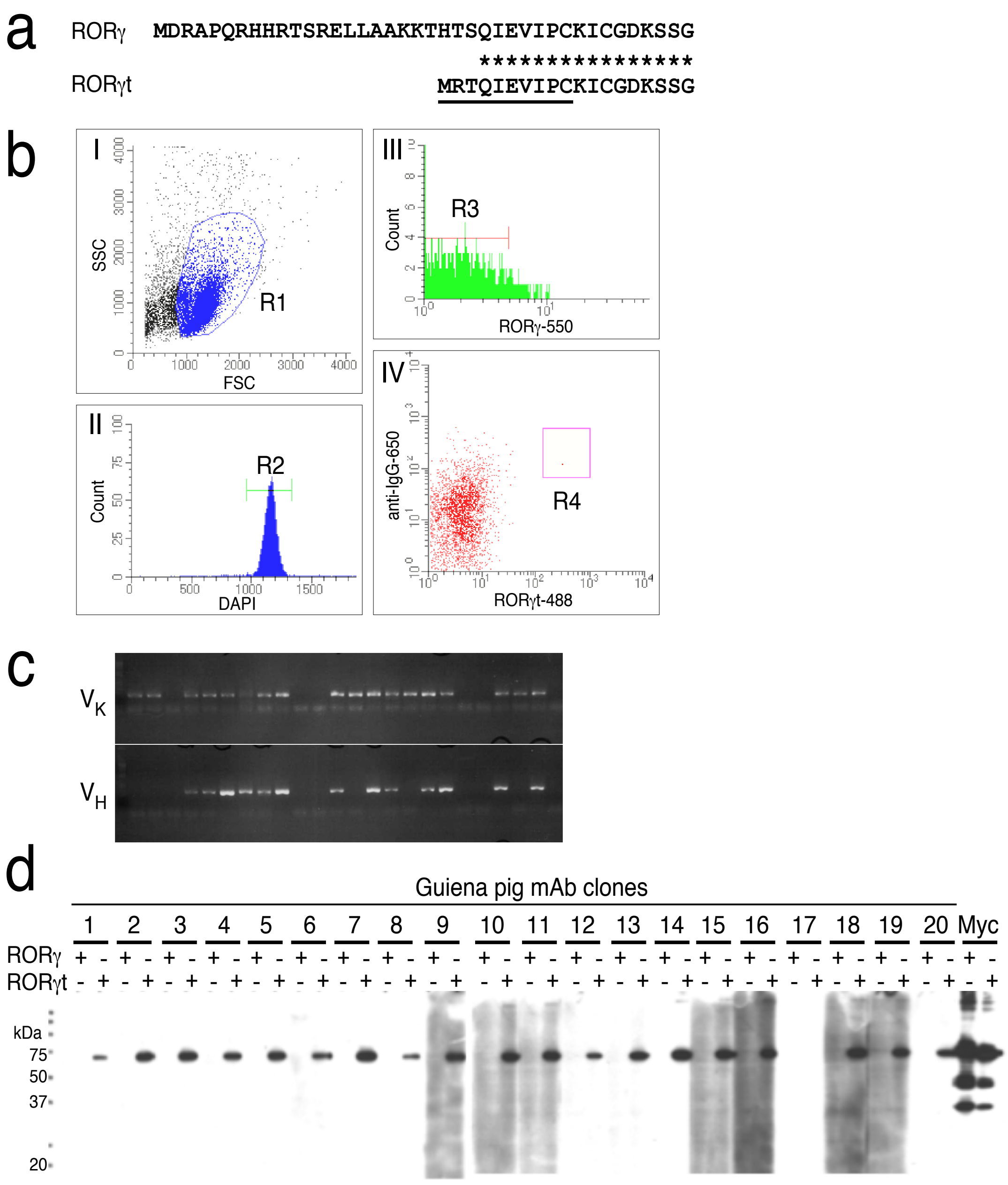

Supplementary figure 2. FIXAA enables to produce mAbs from a variety of animals.

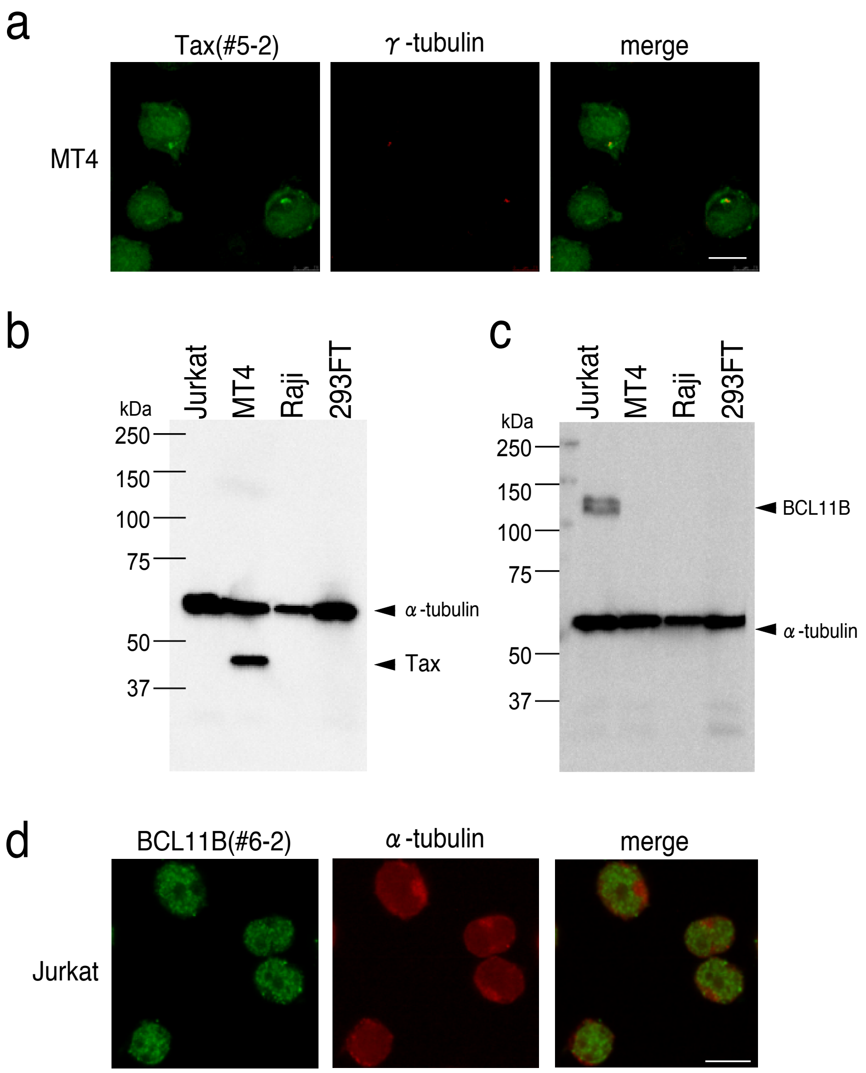

Supplementary figure 3. Generation of a mAb against T68-phosphorylated CHK2.

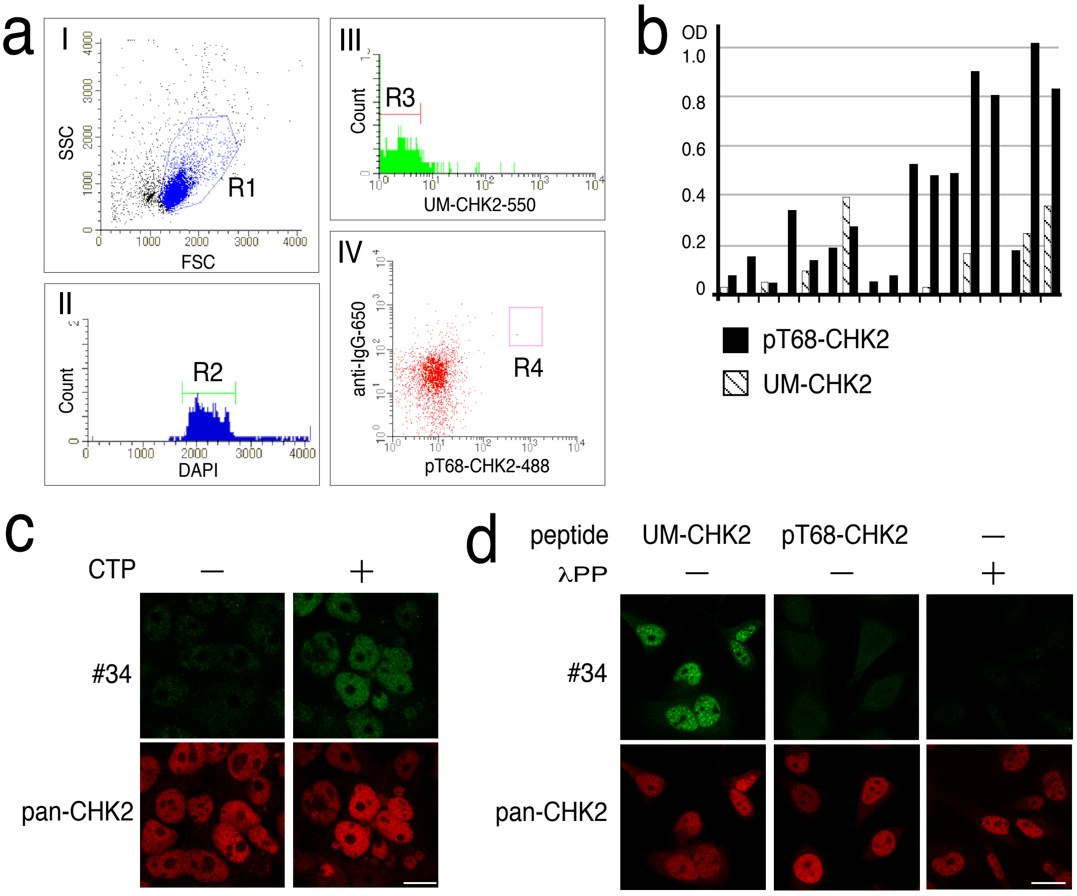

e

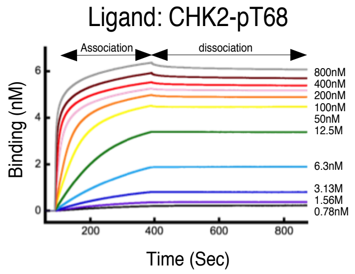

Peptide	K_D (nM)	K_a ($x10^5M^{-1}S^{-1}$)	K_d ($x10^{-3}S^{-1}$)
pT68-CHK2	0.08±0.03	4.6±0.1	0.04±0.01
UM-CHK2	300	0.66	20
